# Achieving Highly Efficient Photoelectrochemical Water Oxidation with a TiCl_4_ Treated 3D Antimony‐Doped SnO_2_ Macropore/Branched α‐Fe_2_O_3_ Nanorod Heterojunction Photoanode

**DOI:** 10.1002/advs.201500049

**Published:** 2015-05-15

**Authors:** Yang‐Fan Xu, Hua‐Shang Rao, Bai‐Xue Chen, Ying Lin, Hong‐Yan Chen, Dai‐Bin Kuang, Cheng‐Yong Su

**Affiliations:** ^1^ MOE Key Laboratory of Bioinorganic and Synthetic ChemistryLehn Institute of Functional MaterialsSchool of Chemistry and Chemical EngineeringSun Yat‐sen UniversityGuangzhou510275P. R. China

**Keywords:** ATO, hematite, heterojunction, macropore, PEC water splitting

## Abstract

Utilizing photoelectrochemical (PEC) cells to directly collecting solar energy into chemical fuels (e.g., H_2_ via water splitting) is a promising way to tackle the energy challenge. α‐Fe_2_O_3_ has emerged as a desirable photoanode material in a PEC cell due to its wide spectrum absorption range, chemical stability, and earth abundant component. However, the short excited state lifetime, poor minority charge carrier mobility, and long light penetration depth hamper its application. Recently, the elegantly designed hierarchical macroporous composite nanomaterial has emerged as a strong candidate for photoelectrical applications. Here, a novel 3D antimony‐doped SnO_2_ (ATO) macroporous structure is demonstrated as a transparent conducting scaffold to load 1D hematite nanorod to form a composite material for efficient PEC water splitting. An enormous enhancement in PEC performance is found in the 3D electrode compared to the controlled planar one, due to the outstanding light harvesting and charge transport. A facile and simple TiCl_4_ treatment further introduces the Ti doping into the hematite while simultaneously forming a passivation layer to eliminate adverse reactions. The results indicate that the structural design and nanoengineering are an effective strategy to boost the PEC performance in order to bring more potential devices into practical use.

## 1 Introduction

Efficiently utilizing solar energy is one of the key procedures to tackle the energy and environmental issues. Compared with the diurnal‐worked solar cells, inspired from the photosynthesis in nature, the technologies such as solar‐driven photoelectrochemical (PEC) water splitting or reduction of CO_2_ whose working principle is to convert solar energy to chemical bonds that can be stored and off‐hour used have provided another anticipated possibility to capture the sunlight.^1^ A lot of materials have been employed as photoelectrodes in PEC cells, among which the α‐Fe_2_O_3_ (also called hematite) is widely acknowledged as a promising candidate due to its earth‐abundant element component, physical and chemical stability, and wide spectrum absorption range in visible light region.^2^ Although the theoretical solar‐to‐hydrogen (STH) efficiency of hematite can reach to 15% if incorporate with a proper tandem photovoltaic (PV) setup,^3^ the performance of the state‐of‐the‐art hematite based photoelectrode is far from the idealized model, owing to the intrinsic adverse factors such as poor electrical conducting ability, restricted hole diffusion length (<5 nm), low absorption coefficient, and low flat‐band potential in water splitting.[[qv: 1b,4]] Much efforts have been devoted to addressing this issues including elemental doping,^5^ morphology tailoring,[[qv: 5e,6]] and surface modification.^7^

Recently, constructing composite photoelectrodes including multicomponent or multiphase heterojunctions have engaged many attentions in designing highly active photocatalyst systems.^8^ Such a host‐guest approach has been proved a valid strategy in enhancing charge carrier separation along with broadening absorption range when compared to the individual host or guest part. Moreover, latest researches on structuring photoelectrodes into novel 3D architectures have shown a great superiority over the planar form in the performance of photovoltaic devices or PEC cells.[[qv: 5d,9]] Such an architecture provides a long optical pathway for substantially harvesting incident photon flux, a considerable specific surface area for absorber (dyes, quantum dots, photocatalysts) loading, and a shortened electron transport route for effective charge collection. All of the positive factors will account for a better solar energy conversion efficiency.

The transparent conducting oxides (TCOs) materials, such as FTO (F:SnO_2_), ATO (Sb:SnO_2_), NTO (Nb:SnO_2_), and AZO (Al:ZnO), uniquely bond excellent electronic conductivity with high transparency in the visible region of the spectrum, perfectly fulfilling the requirements of the host scaffold in constructing composite electrodes for PEC devices. Recently, NTO nanoparticle,^10^ 1D ATO nanorod array,^11^ and 3D photonic FTO nanocone array[[qv: 9g]] have been successfully used as transparent conductive host for loading the hematite nanoparticle or nanoplate guest which exhibit exciting PEC performances. Given that the 1D hematite nanorod owns an enhanced conductivity along [110] axis,[[qv: 2b]] it is predictable that incorporating 1D hematite nanorod with TCOs will lead to an eminent performance.

Herein, for the first time we demonstrate a novel 3D antimony‐doped SnO_2_ (ATO) macroporous structure as transparent conducting scaffolds incorporating with 1D hematite nanorod as composite heterostructure photocatalyst for efficient PEC water splitting. ATO is one of the most frequently studied TCOs materials due to its outstanding chemical stability over a wide range of pH, abundant elemental resources, and low cost.^12^ The elegantly designed 3D heterostructure photoelectrode shows manifest advancement than the 1D hematite nanorod array onto planar FTO glass. With the following TiCl_4_ treatment and the introduction of Co‐Pi co‐catalyst, an impressive photo­current density of 3.27 mA cm^−2^ at 1.23 V versus reversible hydrogen electrode (RHE) is finally achieved.

## 2 Results and Discussions

### 2.1 Fabrication and Characterization of Antimony‐Doped SnO_2_ Macropore/Branched α‐Fe_2_O_3_ Nanorod Heterojunction Photoanodes

**Scheme 1:**
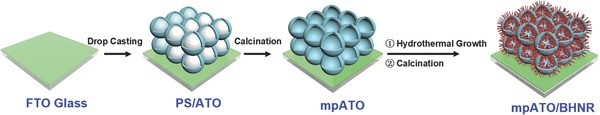
A schematic of the fabrication of the 3D ATO macropore/branched α‐Fe_2_O_3_ nanorod heterojunction photoelectrode.

The experimental details are illustrated in the Experimental Section and diagramed as **Scheme** **1**. First, the macroporous ATO (mpATO) structure was prepared by drop casting the polystyrene (PS)/ATO suspension solution which was synthesized by modified template‐assisted method[[qv: 9b,13]] onto FTO glass and followed an annealing process to remove the PS template. X‐ray diffraction (XRD) patterns (Figure S1a, Supporting Information) illustrate that all samples of various Sb dopant concentrations match well with the SnO_2_ cassiterite phase (JCPDS No. 41‐1445) without any evidenced antimony‐oxide areas, even the doping concentration is up to 15%, which means the Sb acts as a dopant into SnO_2_ lattice rather than segregates out to form another phase.

The field emission scanning electron microscope (FE‐SEM) and transmission electron microscope (TEM) images (Figure S2a–c, Supporting Information) demonstrate that the monodispersed PS sphere templates and ATO hollow spheres with diameter of 400 nm were successfully fabricated. The high resolution transmission electron microscope (HRTEM) image (Figure S2d, Supporting Information) further reveals the ATO hollow sphere is composed of 5 nm nanoparticles and the spacings between the adjacent lattice fringes are 0.3328 and 0.2632 nm, corresponding to (110) and (101) planes of the SnO_2_, while both of which evince a shrink compared to the standard data. The X‐ray photoelectron spectroscopy (XPS) spectrum was measured and analyzed to further confirm the Sb has doped into the SnO_2_. The high‐resolution spectrum (Figure S3a, Supporting Information) probes that oxidation states of Sn is 4+ according to the peaks located at 495.4 eV (3d_3/2_) and 487.29 eV (3d_5/2_). Two peaks at 540.6 and 539.8 eV correspond to the mixed valance states of Sb^5+^ and Sb^3+^, respectively, were deconvoluted out by fitting the Sb 3d_3/2_ peak at binding energy of 540.5 eV. The fitting result reveals that the Sb^5+^ is the major state, representing effective n‐type doping and an elevation of electron conductivity.^12^

**Figure 1:**
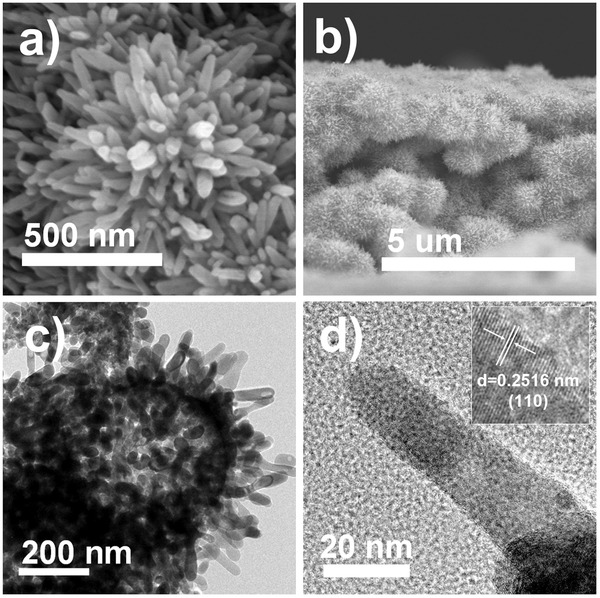
a) Top view FE‐SEM image of 10%‐mpATO/BHNR. b) Cross‐sectional view FE‐SEM image of 10%‐mpATO/BHNR. c,d) The TEM and HRTEM images of the 10%‐mpATO/BHNR.

Hydrothermal reaction was sequentially carried out to introduce FeOOH nanorod epitaxially growing into mpATO film. The XRD patterns (Figure S1b,c, Supporting Information) indicate the as‐synthesized FeOOH nanorod has been transformed to Hematite nanorod (HNR) after thermal treatment. FE‐SEM images (**Figure** **1**a,b) display the top and cross‐sectional views of the 10% Sb doping macroporous antimony‐doped SnO_2_/branched α‐Fe_2_O_3_ nanorod (10%‐mpATO/BHNR) film. Clearly, the dense hematite nanorods epitaxially growing on the surface of ATO macropores to form a 3D architecture, which might be ascribed to the low interfacial lattice mismatch between SnO_2_ and FeOOH.^14^ TEM image (Figure 1c) further shows the hematite nanorod was approximately 120 nm in length and 20 nm in diameter. The clear lattice fringes with spacing of 0.2470 nm in HRTEM images (Figure 1d) agrees well with the (110) plane of the hematite.

### 2.2 Photoelectrochemical Water‐Splitting Performance

To investigate the photoelectric performances of the various mpATO/BHNR film, the photoelectrochemical characterizations were measured in a three‐electrode configuration consisting of Pt mesh counter electrode and Ag/AgCl (in saturated KCl) as reference electrode while the electrolyte was 1 m NaOH (pH = 13.5) solution. A 150 W Xe lamp and AM 1.5G filter were employed to simulate the solar radiation (100 mW cm^−2^). The applied potentials versus reference electrode were converted to the RHE scale according to the Nernst equation

(1) $$E_{\mathrm{RHE}}=E_{\mathrm{Ag/AgCl}}+0.\mathrm{059}\;\mathrm{pH}+E^{0}\mathrm{Ag/AgCl}$$

**Figure 2:**
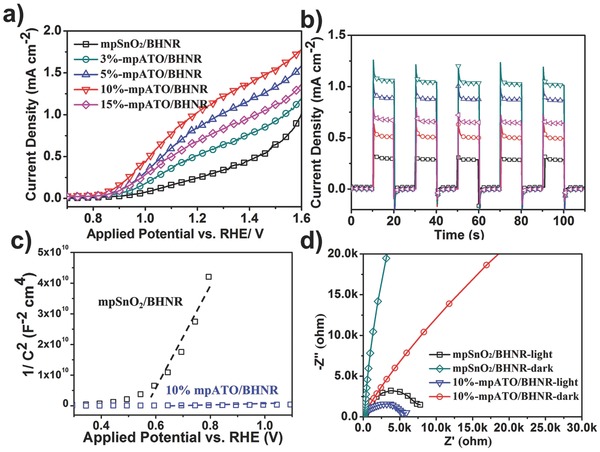
a) Linear sweep voltammograms of mpSnO_2_/BHNR sample and mpATO/BHNR samples with different Sb dopant concentrations under illumination and b) the corresponding amperometric *I*–*t* curves plotted at an external potential of 1.23 V versus RHE, under chopped illumination. c) The Mott–Schottky plots and d) the EIS Nyquist plots of the 10%‐mpATO/BHNR and mpSnO_2_/BHNR.

Where the *E*_RHE_ is the converted potential with respect to RHE, *E*_Ag/AgCl_ is the external potential measured against the Ag/AgCl reference electrode, *E* ^0^_Ag/AgCl_ is the standard potential of Ag/AgCl reference electrode at 25 °C (here is 0.1976 V). First, we devote our efforts on probing the influence of the Sb dopant concentration in mpATO scaffolds on the PEC performance. The linear sweep voltammetry (LSV) data (**Figure** **2**a) collected for the mpSnO_2_/BHNR sample and mpATO/BHNR samples with various Sb concentrations under simulated illumination at a scan rate of 10 mV s^−1^ clearly illustrate the boost in photocurrent density of mpATO/BHNR samples compared to the undoped mpSnO_2_/BHNR one. The photocurrent density finally achieved a vertex when the Sb dopant concentration reached to 10%, which is four times of the undoped one at 1.23 V versus RHE (1.10 mA cm^−2^ vs 0.27 mA cm^−2^). Further increasing dopant concentration to 15% leads to an evident decline of the photocurrent response, which can be ascribed to the drop of conductivity of 15% ATO caused by the detrimental electron scattering effect for the heavy dopant concentration. In principle, the electrical conductivity (*σ*) is defined as

(2) σ=Neμ

Where *N* represents the carrier concentration, *e* represents carrier charge, and *μ* is the mobility.^15^ As for ATO, the presence of Sb^5+^ occupying Sn^4+^ sites leads to the generation of free electrons to maintain charge neutrality, thus increasing the charge carrier concentration. However, too much dopants (e.g., 15%) may cause serious defects and inhibit the movement of carriers through material, which further decrease the mobility and the conductivity. Moreover, all the samples exhibit an onset potential of ≈0.82 V versus RHE, which is in coincidence with the reported values of hematite nanorods in the same test condition.[[qv: 6e,7c]] The amperometric photocurrent density–time (*I*–*t*) curves recorded at 1.23 V external potential versus RHE under chopped illumination (Figure 2b) exhibit the same photoelectrochemical performance trend as the LSV plots. All the samples possess excellent sensitivity to the light illumination and reproducible photocurrent response via several light on‐light off cycles. Both transient photocurrent and steady‐state photocurrent increased for the mpATO/BHNR compared to the undoped mpSnO_2_/BHNR, which means more charges were generated and diffused to the surface of semiconductor and react with the electrolyte.

Mott–Schottky measurements were performed in 1 m NaOH under dark condition to affirm the role of Sb dopant in carrier density of mpATO. According to the Mott‐Schottky equation:

(3) $$N_{d}=(\mathrm{2/}e_{0}\varepsilon_{0}\varepsilon ){\left[d\;(\mathrm{1/}C^{2})dV\right]}^{-1}$$

Where *N*_d_ is the carrier density and *C* is the capacitance derived from the electrochemical impedance obtained at each potential with 10 KHz. *e*_0_, *ε*_0_, *ε* are constants representing electron charge, the dielectric constant of hematite, and the permittivity of vacuum, respectively. A significant improvement in carrier density for mpATO/BHNR can be found from the Mott–Schottky plots by comparing the slope value of the two samples (Figure 2c), while the lower value means higher donor density. The calculated carrier density of 10%‐mpATO/BHNR exhibits a three order of magnitude enhancement than the mpSnO_2_/BHNR, which will largely increase the conductivity, and further contribute to the charge transportation and photocurrent density.

Electrochemical impedance spectroscopy (EIS), which is wildly used in many fields relate to electrochemical behaviors, was tested to further understand the influence of Sb dopant on the conductivity and the charge transfer process. Figure 2d is the EIS spectra of the 10%‐mpATO/BHNR and mpSnO_2_/HNR samples measured at a fixed voltage (1.23 V vs RHE) in dark or under illumination condition. Generally, a smaller radius in the diagram is associated to lower electron transport resistance and higher separation efficiency of the photogenerated electrons and holes.[[qv: 5d,16]] Clearly, the 10%‐mpATO/BHNR performs better than the undoped mpSnO_2_/BHNR due to the smaller radius in the testing range of 100 kHz to 100 mHz. In addition, the curves under illumination exhibit much smaller arch compared to those under the dark conditions, due to the increased electron conductivity under illumination.[[qv: 8b]] Integrate the results from linear sweep voltammograms, amperometric photocurrent density–time (*I*–*t*) curves, Mott–Schottky measurements and EIS spectra, it is our belief that the enhanced conductivity for the mpATO would contribute to the charge separation and transportation, hence responsible for the positive impact on the photoelectrochemical performance.

To investigate the advances of 3D architecture, a similar hydrothermal process was carried out on planar FTO glass to form the FTO/hematite nanorod (FTO/HNR) photoelectrode as controlled sample. FE‐SEM images (Figure S4, Supporting Information) show the length of hematite nanorod array directly grew onto FTO glass is approximately 750 nm. The linear sweep voltammogram of the FTO/HNR (Figure S5a, Supporting Information) reveals a distinct weakness in photoelectrochemical performance with photocurrent density of 0.13 mA cm^−2^ at 1.23 V vs RHE, compared to 1.10 mA cm^−2^ for the 10%‐mpATO/BHNR (Figure 2a). The augment of photocurrent density of 10%‐mpATO/BHNR stems from the shorter electron transport routes and intensive hematite loading. The available surface area for 10%‐mpATO/BHNR evaluated by cyclic voltammetry shows 16 times enhancement than that of FTO/HNR (Figure S6, Supporting Information). The optical characterizations of the two samples (Figure S5b–e, Supporting Information) clearly display that the 10%‐mpATO/BHNR has an enhanced absorption behavior in the visible spectrum range than that of the FTO/HNR, since the macroporous structure owns significant advantages compared to planar FTO glass such as large surface area and excellent light scattering ability.[[qv: 9b]] The enlarged surface area can provide more sites for chemical reactions and the enhanced light scattering ability can prolong the pathway of the incident light in the film for efficiently utilizing the light. The digital photographs (Figure S7, Supporting Information) clearly illustrate that 3D configuration electrodes exhibit a dark and opaque display compare to the planar ones.

### 2.3 The Influence of TiCl_4_ Post Treatment

Introducing substitutional doping elements or forming surface passivating layer are extensively recognized as efficient strategy to enhance the photoelectrochemical performance.^5^, ^7^ Herein we realize the Ti doped hematite and TiO_2_ passivating layer on the surface of hematite nanorod and mpATO by a simple hydrothermal decomposition of TiCl_4_ followed a postannealing process (see the Experimental Section for details).

**Figure 3:**
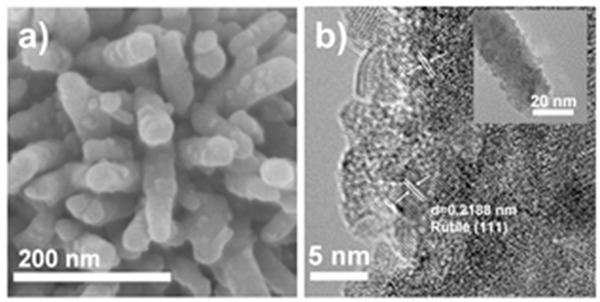
a) High magnification FE‐SEM image and b) HRTEM image of the TiCl_4_ treated 10%‐mpATO/BHNR, inset is the corresponding TEM image.

The high magnification FE‐SEM image of the TiCl_4_ treated 10%‐mpATO/BHNR sample (**Figure** **3**a) clearly shows that the surface of the hematite nanorod becomes rough and is covered by a layer of nanoparticles, which is further confirmed by the TEM observation (inset in Figure 3b). HRTEM image (Figure 3b) depicts that the postintroduced TiO_2_ nanoparticles with 3 nm can initially be recognized as rutile TiO_2_ according to the lattice fringes with spacings of 0.2188 nm.

**Figure 4:**
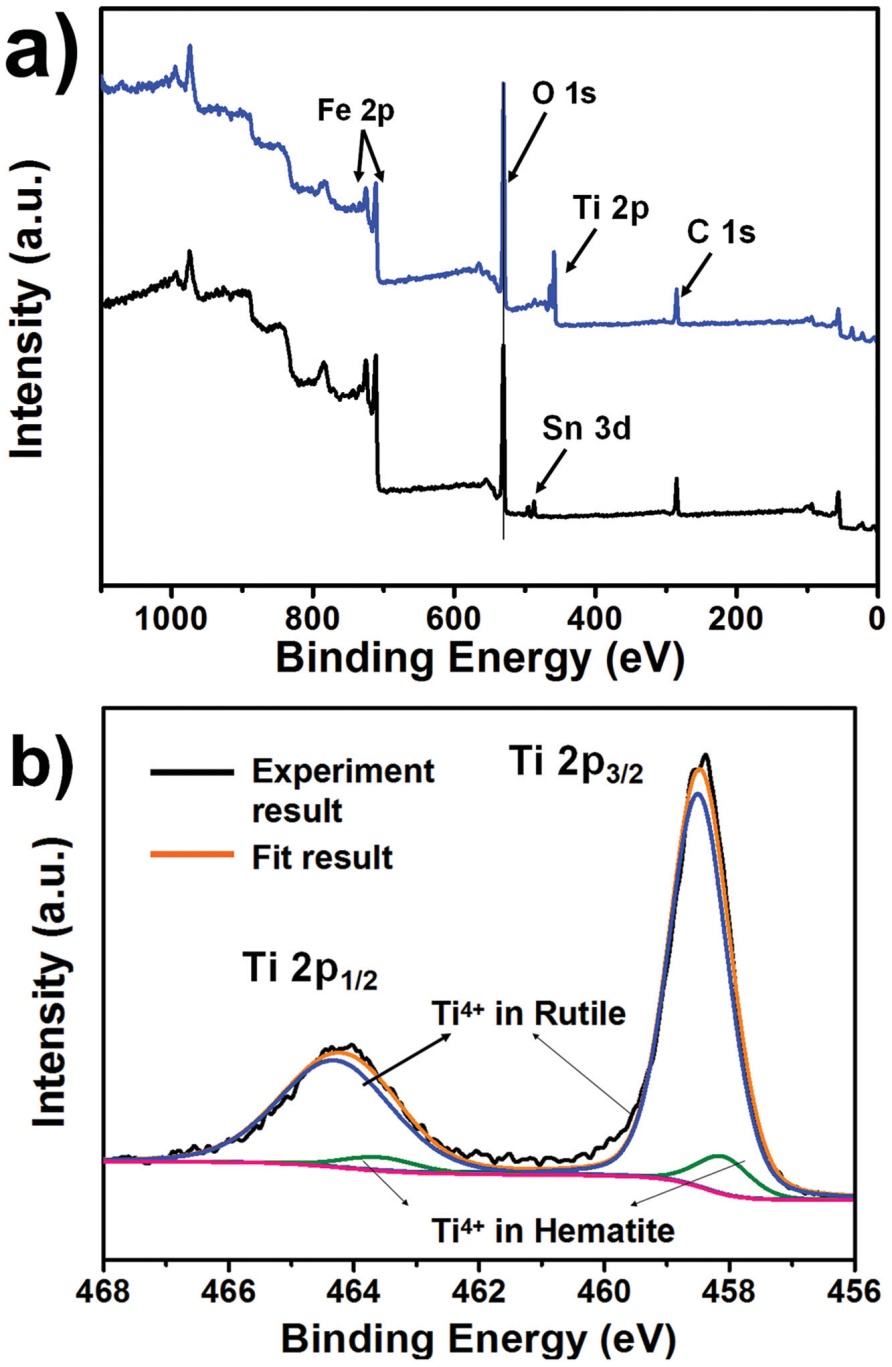
a) XPS survey spectra of the pristine untreated sample (black) and the TiCl_4_ treated one (blue). b) High‐resolution Ti XPS spectra of the TiCl_4_ treated 10%‐mpATO/BHNR sample.

**Figure 5:**
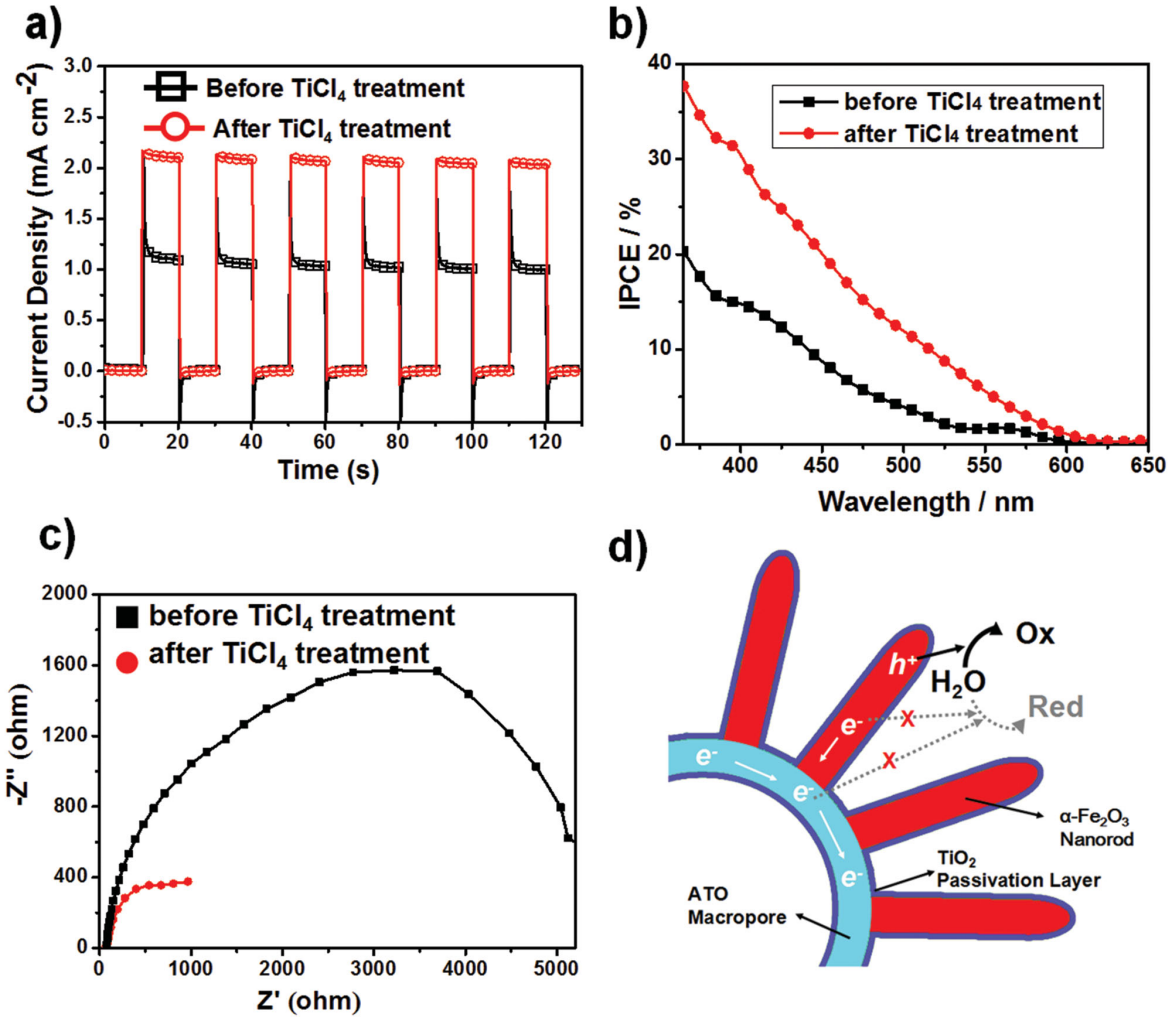
a) The amperometric *I*–*t* curves plotted for the untreated and TiCl_4_ treated 10%‐mpATO/BHNR samples under chopped illumination at 1.23 V versus RHE. b) Incident photon‐to‐current efficiency (IPCE) spectra and c) PEIS plots of the 10%‐mpATO/BHNR before or after TiCl_4_ treatment. d) The sketch of the role of TiCl_4_ treatment.

XPS was carried out to offer more details about the environment of Ti in the surface to investigate the role of the TiCl_4_ treatment. The survey spectrum of the pristine untreated sample, and the TiCl_4_ treated sample are shown in **Figure** **4**a. Compared with the pristine one, the TiCl_4_ treated sample shows an existence of Ti^4+^ according to two main peaks at binding energies between 456 and 468 eV, belonging to Ti 2p_1/2_ and Ti 2p_3/2_. The high resolution XPS spectrum (Figure 4b) reveals that the peak of Ti 2p_3/2_ at binding energy of ≈458.3 eV shows a red shift compared to the reported value for pure TiO_2_ (458.5 eV for 2p_3/2_ binding energy), which elucidates that the Ti^4+^ exists in more than one environment. The fitting results show the Ti 2p_3/2_ peaks can be deconvoluted into two separate peaks with binding energies of 458.5 and 458.1 eV, which correspond to the Ti^4+^ in rutile TiO_2_ and Ti^4+^ doped into hematite, respectively. Mott–Schottky plots (Figure S8a, Supporting Information) further affirm the carrier density was found a 3.5 times enhancement for TiCl_4_ treated sample compared to the pristine one, which is responsible for the enlarged photocurrent density due to the promoted conductivity. The amperometric photocurrent density–time (*I*–*t*) curves were measured to probe the photoresponse and the surface modification of the photoelectrodes after TiCl_4_ treatment. *I*–*t* curves (**Figure** **5**a) delineate a conspicuous variation between the pristine untreated and TiCl_4_ treated ones. First of all, the steady‐state photocurrent density of TiCl_4_ treated sample reached to 2.13 mA cm^−2^ at 1.23 V versus RHE, reflecting a 1.9 times enhancement. Moreover, the transient photocurrent in the untreated one was obvious, however, would almost disappear after the TiCl_4_ treatment. In general, the transient anodic photocurrent is recognized as the consequence of the accumulated photogenerated holes at the semiconductor–electrolyte interface due to the slow oxygen evolution reaction (OER) kinetics^17^ or the carriers oxidize trap states in the bulk^18^ and on surface.^19^ On the contrary, the cathodic transient photocurrent is regarded as electrons diffusing from the external circuit and recombining with the accumulated holes at the interface when turns off the light. A suppressive transient photocurrent for the TiCl_4_ treated sample can be ascribed to the less holes at the semiconductor interface, which is proved by the LSV curves of samples measured in the dark (Figure S8b, Supporting Information). In addition, a negative shift of onset potential represents the electrical oxidation of water to O_2_ was found after the TiCl_4_ treatment, which implies that the surface became more catalytic for O_2_ evolution than the untreated one. On the other hand, the significant recombination caused by the presence of surface states could be eliminated by the formation of metal oxide overlayer, since the commonly existed surface states in nanoscale hematite may serve as the active center to result in serious recombination between the electrons and the accumulated holes.

**Figure 6:**
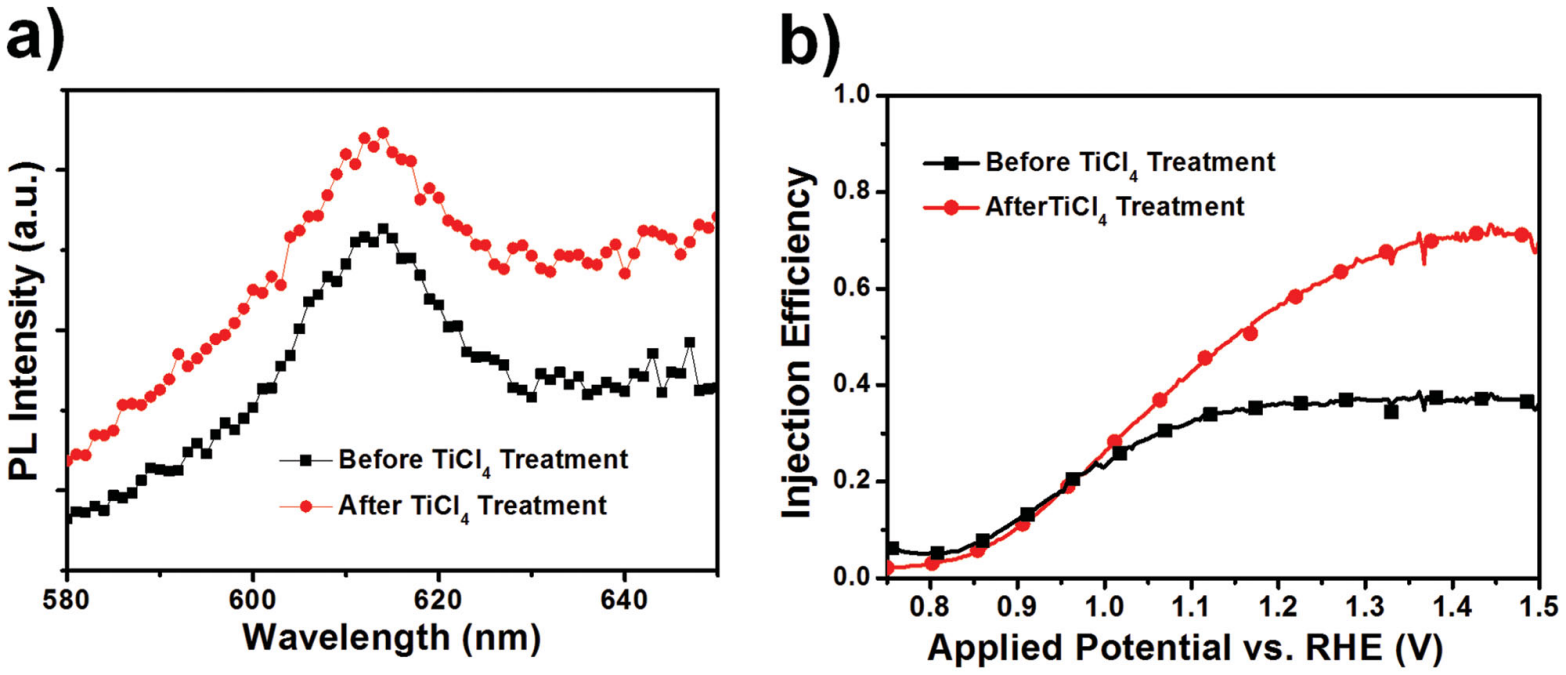
a) The PL emission spectra and b) the calculated injection efficiency of the 10%‐mpATO/BHNR photoanode before and after TiCl_4_ treatment.

The photoluminescence (PL) spectra of the 10%‐mpATO/BHNR photoanode before and after TiCl_4_ treatment (**Figure** **6**a) illustrate that the PL response enhances for the TiCl_4_ treated sample compared with the pristine one, which can be ascribed to the eliminated nonradiative recombination caused by surface trap states.[[qv: 7a,20]] To probe the surface recombination processes and quantitatively evaluate the surface injection efficiency, additional photooxidation tests with the presence of H_2_O_2_ as hole scavenger were carried out,^21^ as shown in Figure 6b and Figure S9, Supporting Information. The TiCl_4_ treated sample performs a higher injection efficiency than the untreated one (61% and 36% at 1.23 V versus RHE, respectively), which further approves that the TiCl_4_ treatment has a profound effect on promoting the surface charge injection and suppressing the surface recombination, leading to less hole accumulation and better photoelectrochemical performance.

It is well known that back reduction on the conductive substrate cannot be neglected, especially when the high aspect‐ratio and conductive TCO materials were involved.^22^ The TiO_2_ blocking layer fabricated by hydrothermal decomposition of TiCl_4_ or electrodeposition has been capable to block the solution‐mediated recombination at the highly conductive substrates.^23^ Hence, the exposed conductive mpATO and FTO glass substrate would be sufficiently covered by the TiO_2_ nanoparticles formed through the current TiCl_4_ treatment, which leads to a declined back reaction and an enhanced photocurrent response.

The incident photon‐to‐electron conversion efficiency (IPCE) spectra performed at 1.23 V versus RHE, as shown in Figure 5b, indicate that the TiCl_4_ treated sample exhibited an enhanced quantum efficiency in the whole test range of 365–650 nm. The IPCE spectra further confirmed the enhanced PEC behavior by the TiCl_4_ treatment.

Photoelectrochemical impedance spectroscopy (PEIS) was again used to probe the charge transfer process at the semiconductor–electrolyte interface. The Nyquist plots (Figure 5c) measured at 1.23 V versus RHE under 100 mW cm^−2^ illumination have shown that a smaller radii of low frequency response for TiCl_4_ treated sample is clearly observed, indicates a better charge transfer process at the semiconductor–electrolyte interface. Such results can be derived from the promoted surface catalytic nature (Figure S8b, Supporting Information) and reduced recombination caused by surface states and back reactions (Figure 5d), which are in accordance with the restrained transient photocurrent density (Figure 5a). Moreover, the stability test of the PEC performance was measured (Figure S10, Supporting Information), which reveals a highly stable photo response for the TiCl_4_ treated 10%‐mpATO/BHNR sample within whole test duration of 1 h.

**Figure 7:**
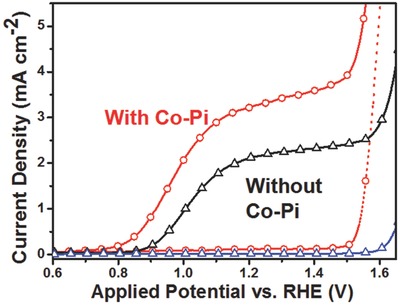
The LSV curves of the TiCl_4_ treated 10%‐mpATO/BHNR sample with or without Co–Pi OER catalyst.

Further optimization by introducing Co–Pi OER catalyst (see the Experimental Section for details) remarkably boosting photocurrent density to 3.27 mA cm^−2^ at 1.23 V versus RHE, along with a significant cathodic shift of onset potential about 100 mV (**Figure** **7**). The impressive photocurrent response is superior or comparable to the reported hematite‐based high performance PEC water splitting systems, which are summarized in Table S1, Supporting Information.

## 3 Conclusion

We have demonstrated a novel and elegant design to incorporate 1D hematite nanorod with conductive 3D macroporous ATO for enhanced conductivity, improved photocatalyst loading, efficient charge transport, and separation. The experiment results evince the well‐grounded design guidelines of high performance PEC cells. Furthermore, a simple and facile TiCl_4_ treatment was employed to introduce the Ti doping into the hematite to promote the conductivity and simultaneously forming the TiO_2_ nanoparticle passivating layer on the surface of hematite and mpATO which eliminate the surface states and back reaction recombination, as illustrated in Figure 5d, hence contributing to the significant enhancement of the photocurrent. Finally, an impressive photocurrent density of 3.27 mA cm^−2^ at 1.23 V versus RHE was obtained for the antimony‐doped SnO_2_ macropore/branched α‐Fe_2_O_3_ nanorod heterojunction photoelectrode. Our effort points out that the structural designing and nanoengineering is an effective strategy to boost the PEC performance in order to make more potential device into practical use.

## 4 Experimental Section

*Preparation of the ATO Macroporous Structure (mpATO)*: The antimony‐doped SnO_2_ (ATO) macropore was synthesized by a template assisted method. First, the monodispersed PS latex solution with 400 nm in diameter were synthesized through an emulsifier‐free reaction.^24^ To prepare the ATO coated PS spheres (PS/ATO), a modified hot reflux method was employed.^13^ Typically, 10 mL H_2_O and 10 mL PS latex (approximately 0.1 g mL^−1^) were mixed in the 100 mL round bottom flask with vigorously stirring. The mixed solution were then settled in the oil bath and heated to 100 °C. Then 10 mL 0.1 m SnCl_2_ ethanol solution and 10 mL SbCl_3_ ethanol solution were controlled, respectively, and simultaneously dropwise adding to the refluxed PS solution by two drop funnel. The reaction was kept for another 12 h and naturally cooled down to room temperature. The precipitates were collected and re‐dispersed in absolute ethanol after tautologically washing procedure. The Sb doping concentrations can be easily tuned by adjusting SbCl_3_ amount. A simple drop casting method similar to our previous report[[qv: 9a]] was employed to form a PS/ATO thin film onto the FTO glass substrate and subsequently annealed in the box furnace at 500 °C for 1 h with heating ramp of 2 °C min^−1^ to remove the PS sphere templates and finally got the macroporous ATO film on FTO glass substrate.

*Preparation of the ATO Macropore*/*Branched α‐Fe_2_O_3_ Nanorod Structure (mpATO/BHNR)*: Typically, 1 mmol FeCl_3_·6H_2_O and 1 mmol Na_2_SO_4_ were dissolved into 25 mL deionized water to form a transparent solution in a 50 mL Teflon‐lined stainless steel autoclave. Then the as‐prepared FTO/macroporous ATO film were placed in the autoclave with the ATO film side facing down. The hydrothermal synthesis was maintained at 120 °C for 5 h and was air‐cooled to room temperature naturally. The as‐prepared macroporous ATO/FeOOH nanorod film was rinsed with water, ethanol and dried at 70 °C. The film was annealed in the box furnace at 550 °C for 2 h to decompose the FeOOH to α‐Fe_2_O_3_ (hematite) and then heated at 600 °C for 30 min to activate the hematite.

*Preparation of α‐Fe_2_O_3_ Nanorod Array onto FTO Glass (FTO*/*HNR)*: A similar hydrothermal procedure was carried out as aforementioned except the FTO/macroporous ATO film were replaced by the bare FTO glass. The as‐prepared film was also annealed at 550 °C for 2 h and 600 °C for 30 min to form hematite and activate its photoelectrochemical performance.

*TiCl_4_ Post Treatment*: Inspired by the similar widely used post treatment in assembling dye‐sensitized solar cells (DSSCs) or quantum dot‐sensitized solar cells (QDSSCs), the TiCl_4_ treatment was employed to further enhance the PEC behavior. Typically, the mpATO/BHNR electrode was soaked in the 40 × 10^−3^ m TiCl_4_ solution at 70 °C for 1 h to carry out the hydrolysis reaction. Then the film was heated to 600 °C for 30 min by using a hot air gun.

*Electrodepositon of Co–Pi Catalyst*: Co–Pi OER catalyst was electrodeposited onto the photoelectrode with a modified methods^25^ under constant voltage. Typically, the TiCl_4_‐treated mpATO/BHNR electrode was immersed into 0.2 m phosphorate buffer solution (pH = 7.0) with exposed area of 0.12 cm^−2^, a Pt mesh was used as the counter electrode and an Ag/AgCl (in saturated KCl) electrode was used as the reference electrode. The electrodepositing process was carried out at a constant voltage of 1.1 V versus Ag/AgCl electrode for 45 min.

*Characterizations*: The morphology and composition of the samples were measured using FE‐SEM (JSM‐6330F) and HRTEM (JEOL‐2010 HR). The XRD patterns were recorded on a Bruker D8 Advance X‐ray diffractometer. The XPS were characterized by a photoelectron spectrometer (ESCALAB 250, Thermo Fisher Scientific). A UV–vis–NIR spectrophotometer (Shimadzu UV‐3600) was employed to measure the UV–vis adsorption spectra. Photoluminescence experiments were performed on a photoluminescence spectrometer (FLS980, Edinburgh Instruments Ltd.) with an excitation wavelength of 520 nm. The emission spectra were scanned from 550 to 650 nm with 1 nm increments and a 1 s integration time.

*Electrochemical Characterization*: A Zennium electrochemical workstation (ZAHNER) operated with the Thales 2.29 software was equipped to conduct all the electrochemical characterizations. Photoelectrochemical measurements were performed with 1 m NaOH electrolyte in a standard three‐electrode configuration that the mpATO/BHNR film as the photoanode, a Pt mesh as the counter electrode and an Ag/AgCl (in saturated KCl) electrode as the reference electrode. A 150 W Xe lamp (LSXS‐150, ZOLIX Instruments) coupled with an AM 1.5G filter (BCF‐AM 1.5G‐050, Beijing Zolix Instruments Co. Ltd.) was employed to simulate the sunlight illumination. The light density was adjusted to 100 mW cm^−2^ by calibrating with a standard Si solar cell. The EIS was carried out in the range of 10 mHz to 1 MHz, with AC voltage amplitude of 10 mV at a DC bias of 1.23 V versus RHE in 1 m NaOH electrolyte. The Mott–Schottky measurements were performed at 1 KHz with voltage amplitude of 10 mV under dark condition. IPCE spectra were performed in the same cell system as a function of wavelength from 365 to 650 nm by Zahner Zennium C‐IMPS system (TLS‐03), under a bias at 1.23 V versus RHE. Cyclic voltammetry was performed to study the effect of surface available for chemical reaction according to the published methods.[[qv: 11a,26]]

## Supporting information

As a service to our authors and readers, this journal provides supporting information supplied by the authors. Such materials are peer reviewed and may be re‐organized for online delivery, but are not copy‐edited or typeset. Technical support issues arising from supporting information (other than missing files) should be addressed to the authors.

Supplementary
