# Supplementary material for: Achieving Highly Efficient Photoelectrochemical Water Oxidation with a TiCl4 Treated 3D Antimony‐Doped SnO2 Macropore/Branched α‐Fe2O3 Nanorod Heterojunction Photoanode
Source: Adv Sci (Weinh). 2015 May 15;2(7):1500049. doi: 10.1002/advs.201500049 (PMC5115430; doi:10.1002/advs.201500049)
Supplement: Supplementary file 1 — Supplementary [file ADVS-2-0j-s001.pdf]

Copyright WILEY-VCH Verlag GmbH & Co. KGaA, 69469 Weinheim, Germany, 2015.

ADVANCED SCIENCE
Open Access

Supporting Information

for *Adv. Sci.*, DOI: 10.1002/advs.201500049

Achieving Highly Efficient Photoelectrochemical Water Oxidation with a $TiCl_4$ Treated 3D Antimony-Doped SnO_2 Macropore/Branched α-Fe_2O_3 Nanorod Heterojunction Photoanode

Yang-Fan Xu, Hua-Shang Rao, Bai-Xue Chen, Ying Lin, Hong-Yan Chen, Dai-Bin Kuang, and Cheng-Yong Su*

Copyright WILEY-VCH Verlag GmbH & Co. KGaA, 69469 Weinheim, Germany, 2013.

Supporting Information

Achieving Highly Efficient Photoelectrochemical Water Oxidation with $TiCl_4$ Treated Three-Dimensional Antimony-Doped SnO_2 Macropore/Branched α-Fe_2O_3 Nanorod Heterojunction Photoanode

*Yang-Fan Xu, Hua-Shang Rao, Bai-Xue Chen, Ying Lin, Hong-Yan Chen, Dai-Bin Kuang, * and Cheng-Yong Su*

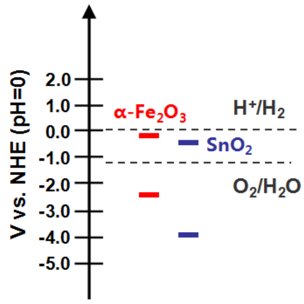

Scheme S1. Schematic presentation of band level positions for α-Fe_2O_3 and SnO_2 based upon existing published data.[1-3]

Based upon the published data, [1-3] the approximate band diagram of the SnO_2/Fe_2O_3 was shown as Scheme S1. According to the band alignment, the SnO_2/hematite composite forms a type II heterojunction which the excited electrons in Fe_2O_3 are thermodynamically admissible to transfer to SnO_2. Moreover, SnO_2 is widely acknowledged as a commercial transparent conducting oxide (TCO) candidate due to its high conductivity and high transparency in the visible region. [4] Besides, Sb-doped SnO_2 (ATO) was carried out to increase its conductivity to further improve the PEC performance.

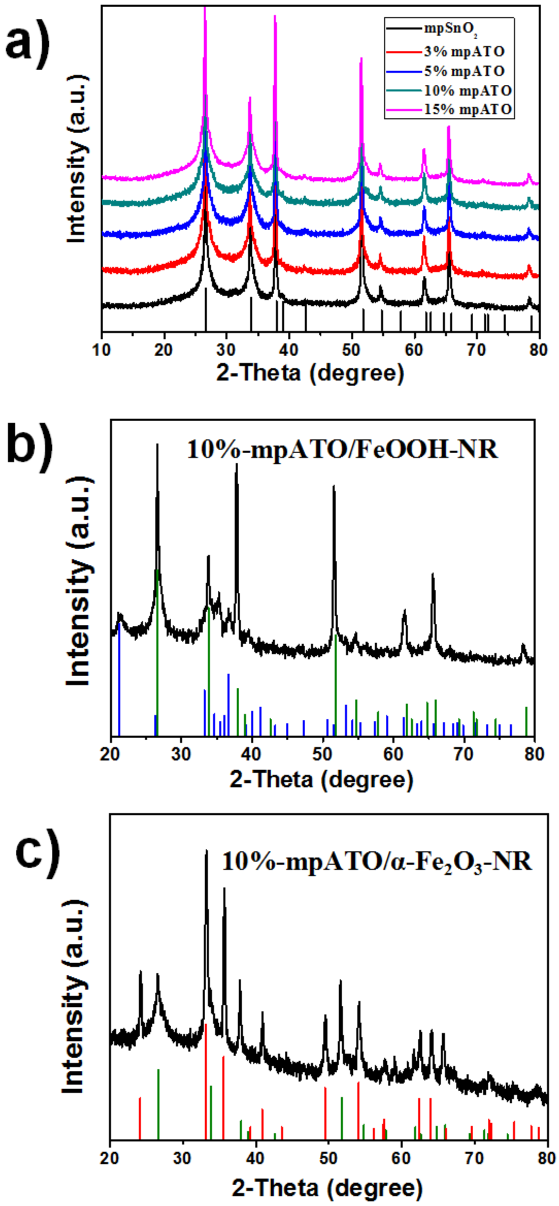

Figure S1. (a) XRD patterns of mpATO with different Sb dopant concentrations, (b) XRD patterns of the 10%-mpATO/FeOOH-NR, the green line and blue line each represents the standard peak of SnO_2 (JCPDS No. 41-1445) and FeOOH (JCPDS No. 29-0713) respectively. (c) XRD patterns of the 10%- mpATO/Fe_2O_3-NR, the green line and red line each represents the standard peak of SnO_2 (JCPDS No. 41-1445) and hematite (JCPDS No. 33-0664) respectively.

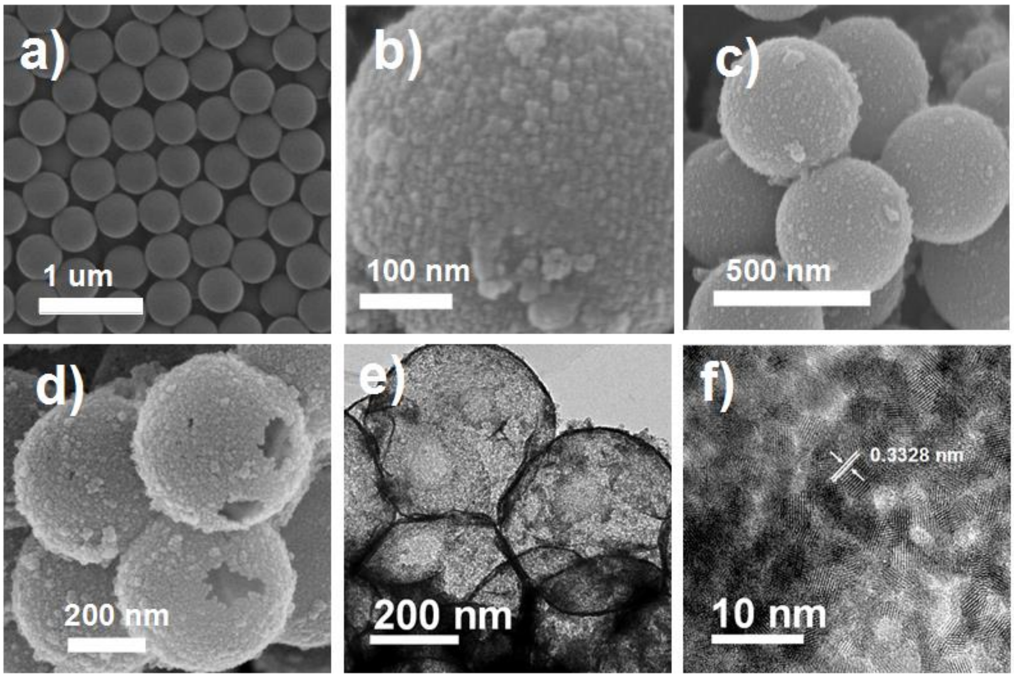

Figure S2. (a) The SEM image of mono-dispersed polystyrene spheres which served as the sacrificial templates. (b, c) The SEM image of the as-prepared PS/10%-ATO beads. (d) The SEM image of the as-annealed 10%-ATO macropores and (d, e) the corresponding TEM and HRTEM images.

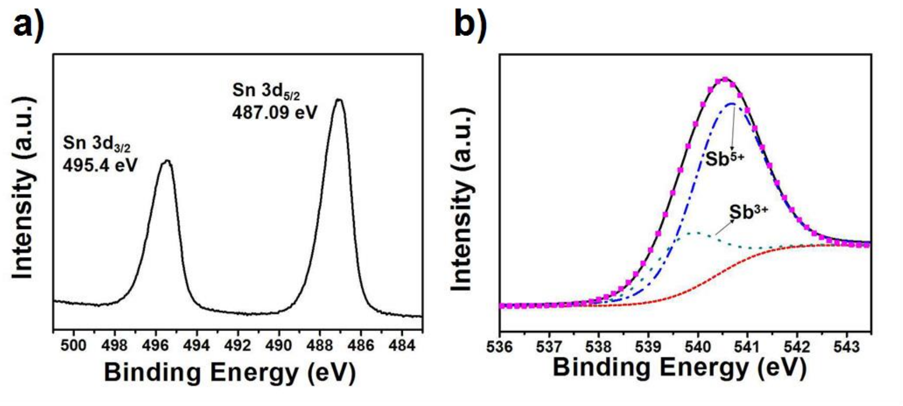

Figure S3. The high-resolution XPS spectroscopy of the core level regions of (a) Sn 3d and (b) Sb $3d_{3/2}$ for the as-prepared 10%-mpATO sample. The deconvoluted peaks of Sb^{5+} ($3d_{3/2}$) and Sb^{3+} ($3d_{3/2}$) are given. The broken red line is the background.

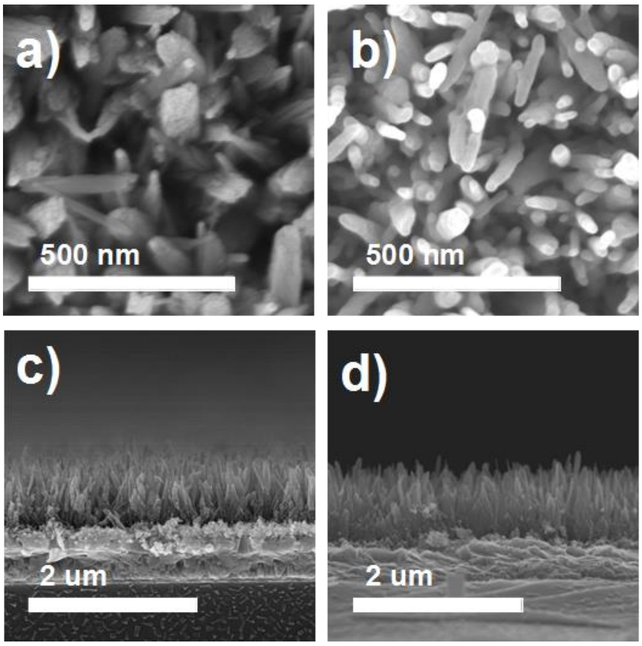

Figure S4. (a) Top view FE-SEM image of the hydrothermal grown FeOOH nanorods onto planar FTO glass. (b) Top view FE-SEM image of FTO/HNR after annealing. The cross-sectional view FE-SEM images of (c) FTO/FeOOH-NR and (d) FTO/HNR further indicate the film thickness did not own a remarkable change.

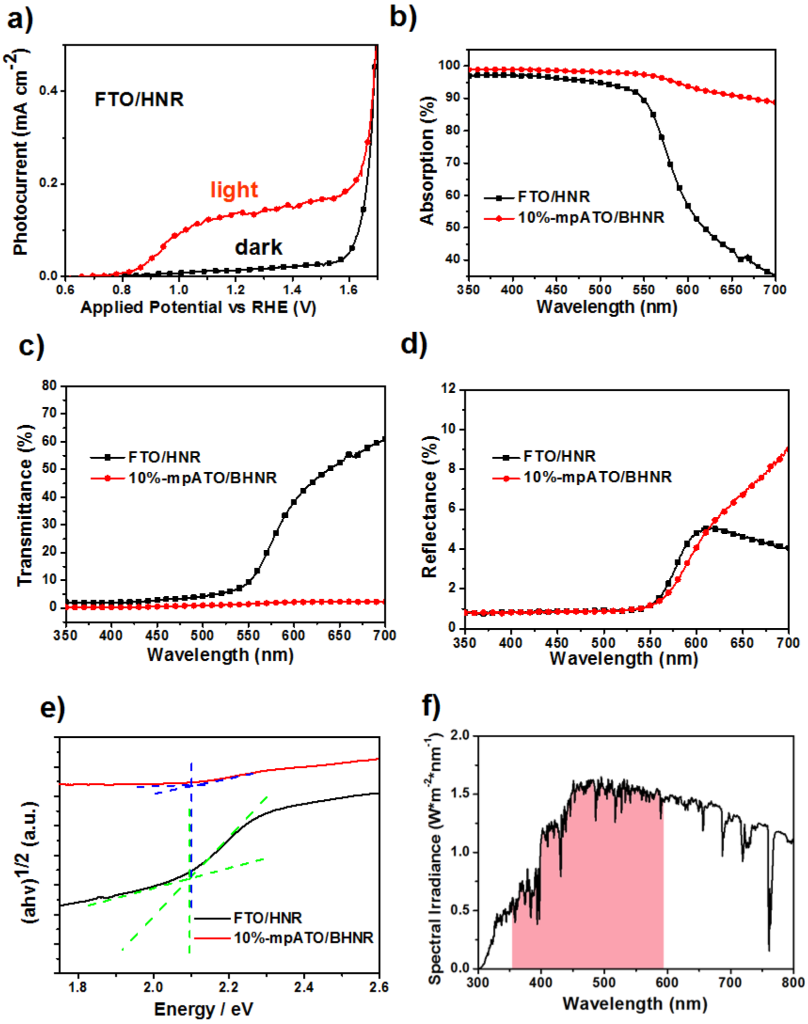

Figure S5. (a) The LSV plots of the FTO/HNR sample under illumination and dark condition in 1 M NaOH electrolyte. The optical characterizations of 10%-mpATO/BHNR and FTO/HNR: (b) absorption spectra, (c) transmittance spectra, (d) reflectance spectra and (e) Tauc plots. (f) The Photon flux of the AM 1.5 G spectrum at 1000 W m^{-2} (ASTM G173-03), the shaded areas is the integration range (350-590 nm).

The absorption (A%) of samples can be calculated according to the formula: A%=1-T%-R%.[5, 6] The plots shown in Figure S5b illustrate that both the FTO/HNR and 10%-mpATO/BHNR possess good absorption behavior in the short wavelength region (< 500 nm) and a significant enhancement in A% can be found in the longer wavelength region (500 nm-600 nm) for the 10%-mpATO/BHNR sample. Moreover, the Tauc plots show that the bandgap of hematite is approximately 2.1 eV, which is in accordance with other reports. By integrating the A% with respect to the AM 1.5G solar spectrum, we can get the $J_{absorbed}$, which is the photon absorption expressed as a current density. The integrated $J_{absorbed}$ over the wavelength range from 350 to 590 nm (*i.e.*, bandgap absorption) is 9.994 mA cm^{-2} and 11.156 mA cm^{-2} for the FTO/HNR and 10%-mpATO/BHNR, respectively. The increased $J_{absorbed}$ can be ascribed to the large surface area for hematite loading and excellent light scattering ability for the 3D macroporous structure.

The measured $J_{photocurrent}$ of the FTO/HNR and 10%-mpATO/BHNR are 0.13 mA cm^{-2} and 1.10 mA cm^{-2} (as shown in Figure 1a and Figure S5a), respectively. The enhancement of $J_{photocurrent}$ for 10%-mpATO/BHNR compared to the FTO/HNR can be ascribed to the following issues: 1) the increased light absorption, 2) ATO macroporous scaffold facile the carrier separation and transportation, 3) as discussed in the manuscript, the hematite nanorod grown in 3D ATO macroporous structure shows a smaller length and width decreasing the diffusion length of carriers, which

makes the hole easily to reach to the semiconductor-electrolyte interface to participate the oxygen evolution reaction (OER) and makes the excited electrons be collected more efficiently.

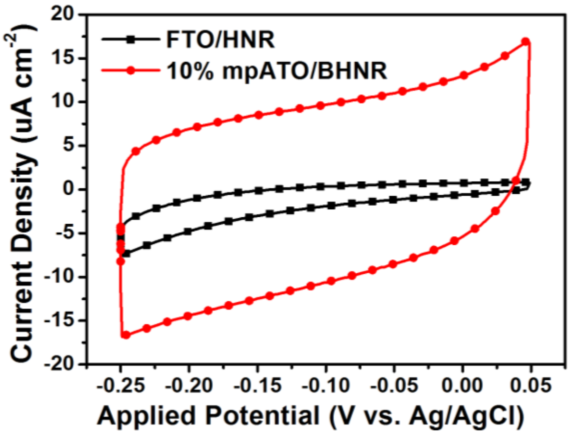

Figure S6. C-V measurement of FTO/HNR and 10%-mpATO/BHNR in 1 M KOH at a scan rate of 50 mV/s.

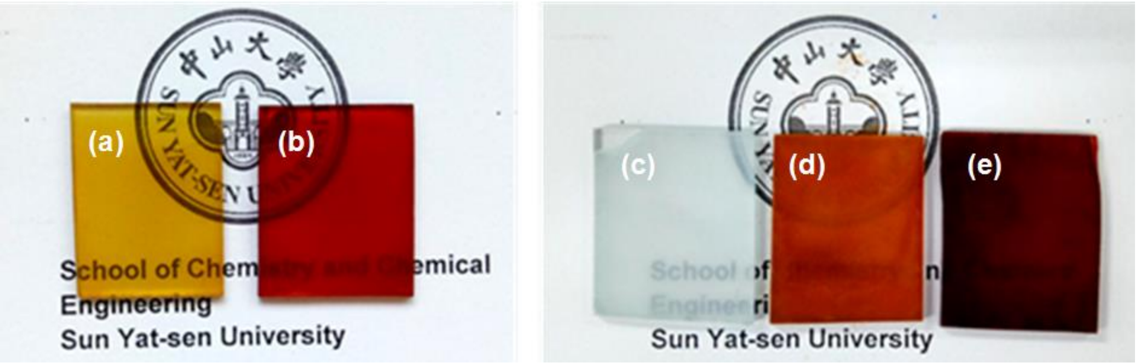

Figure S7. The digital photographs of the photoelectrodes: (a) as-prepared FTO/FeOOH-NR, (b) FTO/HNR, (c) 10%-mpATO, (d) 10%-mpATO/FeOOH-NR and (e) 10%-mpATO/BHNR.

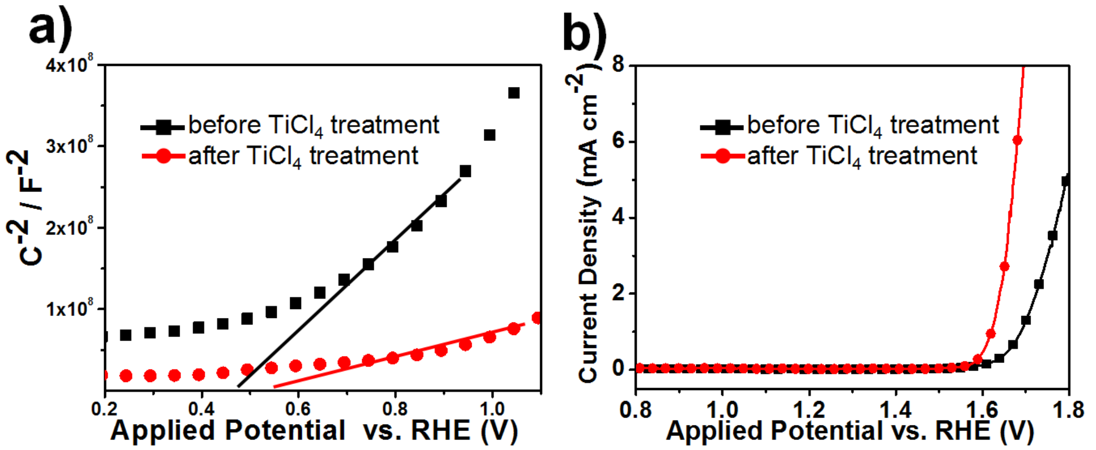

Figure S8. (a) The Mott-Schottky plots and (b) LSV curves (measured in dark condition) of the 10%-mpATO/BHNR before or after $TiCl_4$ treatment.

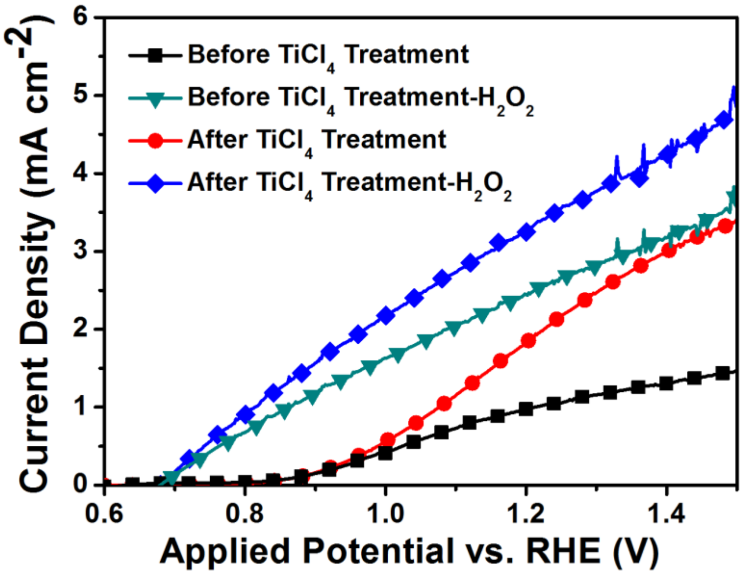

Figure S9. The obtained LSV curves measured in 1 M NaOH or 1 M NaOH + 0.5 M H_2O_2 of 10%-mpATO/BHNR before and after $TiCl_4$ treatment.

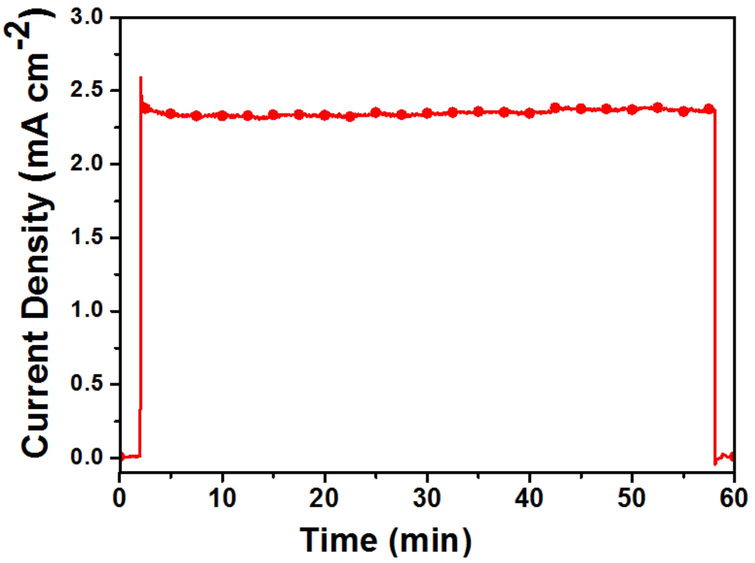

Figure S10. Photocurrent density *vs.* time curves of the $TiCl_4$ treated 10%-mpATO/BHNR for an hour, showing a good stability in the whole test duration.

Table S1. Summary of the PEC performances of hematite-based photoelectrodes.

Hematite morphology	Substrate	Preparation method	Test condition	Current (mA cm^{-2})	Ref.
nanospike	3D FTO	Ultrasonic spray pyrolysis (USP)	1.23 V_{RHE}, 1 M NaOH	3.39	7
nanotree	FTO	Atmospheric Pressure Chemical Vapor Deposition (APCVD)	1.53 V_{RHE}, 1 M NaOH	4.01	8
nanorod	FTO	Hydrothermal method	1.23 V_{RHE}, 1 M NaOH	1.82	9
nanoparticle	ATO nanorods	Electrodeposition	0.6 V_{RHE}, 1 M NaOH	0.67	10
nanoparticle	Nb:SnO 2 NPs	Atomic Layer Deposition (ALD)	1.23 V_{RHE}, 1 M NaOH	2.35	11

References:

[1] M. Gratzel, *Nature* **2001**, *414*, 338.

[2] R. Marschall, *Adv. Funct. Mater.* **2014**, *24*, 2421.

[3] M. Niu, F. Huang, L. Cui, P. Huang, Y. Yu, Y. Wang, *ACS Nano* **2010**, *4*, 681.

[4] R. M. Pasquarelli, D. S. Ginley, R. O'Hayre, *Chem. Soc. Rev.* **2011**, *40*, 5406.

[5] J. Li, Y. Qiu, Z. Wei, Q. Lin, Q. Zhang, K. Yan, H. Chen, S. Xiao, Z. Fan, S. Yang, *Energy Environ. Sci.* **2014**, *7*, 3651.

[6] I. S. Cho, Z. B. Chen, A. J. Forman, D. R. Kim, P. M. Rao, T. F. Jaramillo, X. L. Zheng, *Nano Lett.* **2011**, *11*, 4978.

[7] J. Li, Y. Qiu, Z. Wei, Q. Lin, Q. Zhang, K. Yan, H. Chen, S. Xiao, Z. Fan, S. Yang, *Energy Environ. Sci.* **2014**, *7*, 3651.

[8] S. C. Warren, K. Voitchovsky, H. Dotan, C. M. Leroy, M. Cornuz, F. Stellacci, C. Hebert, A. Rothschild, M. Graetzel, *Nat. Mater.* **2013**, *12*, 842.

[9] Y. C. Ling, G. M. Wang, J. Reddy, C. C. Wang, J. Z. Zhang, Y. Li, *Angew. Chem. Int. Ed.* **2012**, *51*, 4074.

[10] Y. Q. Sun, W. D. Chemelewski, S. P. Berglund, C. Li, H. C. He, G. Q. Shi, C. B. Mullins, *ACS Appl. Mater. Interfaces* **2014**, *6*, 5494.

[11] M. Stefik, M. Cornuz, N. Mathews, T. Hisatomi, S. Mhaisalkar, M. Gratzel, *Nano Lett.* **2012**, *12*, 5431.
